# Role of adipocyte-derived lipoprotein lipase in adipocyte hypertrophy

**DOI:** 10.1186/1743-7075-4-22

**Published:** 2007-10-30

**Authors:** Amanda M Gonzales, Robert A Orlando

**Affiliations:** 1 Department of Biochemistry and Molecular Biology, University of New Mexico, School of Medicine, MSC08 4670, 1 University of New Mexico, Albuquerque, New Mexico, 87131, USA

## Abstract

**Background:**

A major portion of available fatty acids for adipocyte uptake is derived from lipoprotein lipase (LPL)-mediated hydrolysis of circulating lipoprotein particles. In vivo studies aimed at identifying the precise role of adipocyte-derived LPL in fat storage function of adipose tissue have been unable to provide conclusive evidence due to compensatory mechanisms that activate endogenous fatty acid synthesis. To address this gap in knowledge, we have measured the effect of reducing adipocyte LPL expression on intracellular lipid accumulation using a well-established cultured model of adipocyte differentiation.

**Methods:**

siRNA specific for mouse LPL was transfected into 3T3-L1 adipocytes. Expression of LPL was measured by quantitative real-time PCR and cell surface-associated LPL enzymatic activity was measured by colorimetric detection following substrate (p-nitrophenyl butyrate) hydrolysis. Apolipoprotein CII and CIII expression ratios were also measured by qRT-PCR. Intracellular lipid accumulation was quantified by Nile Red staining.

**Results:**

During differentiation of 3T3-L1 pre-adipocytes, LPL mRNA expression increases 6-fold resulting in a 2-fold increase in cell surface-associated LPL enzymatic activity. Parallel to this increase in LPL expression, we found that intracellular lipids increased ~10-fold demonstrating a direct correlation between adipocyte-derived LPL expression and lipid storage. We next reduced LPL expression in adipocytes using siRNA transfections to directly quantify the contributions of adipocyte-derived LPL to lipid storage, This treatment reduced LPL mRNA expression and cell surface-associated LPL enzymatic activity to ~50% of non-treated controls while intracellular lipid levels were reduced by 80%. Exogenous addition of purified LPL (to restore extracellular lipolytic activity) or palmitate (as a source of free fatty acids) to siRNA-treated cells restored intracellular lipid levels to those measured for non-treated controls. We also found that adipocytes express apolipoprotein CII and CIII and, in addition, the apoCII/apoCIII ratio remains largely unchanged in cells with reduced LPL expression.

**Conclusion:**

We provide evidence that adipocyte-derived LPL is required for efficient fatty acid uptake and storage, and that adipocytes express their own source of apoCII and apoCIII for regulating extracellular LPL activity. These findings demonstrate that adipocytes are capable of producing the necessary enzymatic components and co-factors for efficient lipid storage independent of vascular sources.

## Background

The adipocyte plays a crucial role in metabolic regulation, serving as a storage depot for fatty acids and as an endocrine cell to manage energy utilization and feeding behavior [1,2]. The mass of adipose tissue is maintained by a well-controlled balance of cell proliferation (hyperplasia) and increase in fat cell size (hypertrophy). Increases in adipocyte hypertrophy result from the uptake and assimilation of extracellular fatty acids into cytosolic triacylglycerol-rich lipid droplets. The primary sources of these extracellular fatty acids are those that are 1) associated with circulating albumin or 2) hydrolyzed from triacylglycerol-rich lipoprotein particles such as chylomicrons or very low density lipoproteins (VLDL). Since chylomicrons are short-lived fatty acid carriers present only during the post-prandial period, it is accepted that VLDL particles represent the major source of circulating fatty acids in the form of triacylglycerols. Triacylglycerols are the major component of VLDL and provide a concentrated source of esterified fatty acids for peripheral tissues.

The availability of VLDL-derived fatty acids for energy needs and adipocyte storage is dependent upon hydrolytic activity of lipoprotein lipase (LPL) [3-6], and to a lesser extent, endothelial lipase [7,8]. To date, two models have been proposed describing how LPL mediates lipolysis of circulating lipoproteins to release fatty acids for adipocyte uptake [5]. In the first model, LPL originating in adipose or muscle tissue is transported across the endothelial barrier where it remains tethered to the lumenal surface through its affinity for heparan sulfate proteoglycans (HSPG). In this position, LPL can mediate release of fatty acids from circulating lipoproteins for diffusion-based transport to the adipocyte surface or newly released fatty acids are transported through their association with circulating albumin. Alternatively, largely intact lipoproteins can cross the capillary endothelial barrier by either transcytosis [9-11] or through leaky cell-cell junctions. Lipolysis itself is known to cause capillary leakage and can contribute to transendothelial transport [12,13].

Lipoprotein particles that are transported out of the vasculature represent a significant source of fatty acids for adipocyte storage. The release of fatty acids from these particles is likely to be dependent on adipocyte-derived LPL activity. One of our recent studies provides evidence that HSPG found on the surface of adipocytes are essential for lipid accumulation in adipocytes [14]. Although the exact role played by these HSPG toward the uptake of fatty acids remains to be identified; we have hypothesized that HSPG serve as reaction centers for LPL-mediated triacylglycerol hydrolysis. Cell surface HSPG are known to provide high affinity binding sites for both LPL [15,16] and apoE-enriched lipoproteins [17-19]. In this manner, HSPG can serve to co-localize enzyme and substrate, creating a focal reaction center to bring about the release of fatty acids in proximity of the adipocyte cell surface. Uptake of these liberated fatty acids can then be accomplished by fatty acid transport proteins, such as FAT/CD36 [20] or FATP1 [21], which are known to be expressed by adipocytes [22-24]. A significant advantage of this mechanism is that lipoproteins, such as VLDL, can provide a concentrated supply of fatty acids to adipocytes for uptake as opposed to simply relying on free diffusion across the endothelial barrier.

An essential element necessary for this mechanism to be effective is adipocyte-derived LPL activity [25-27]. Studies examining the effects of LPL gene ablation in mice have attempted to define the role of adipocyte-derived LPL in fat storage function of adipose tissue [28]. Homozygous LPL deficiency was found to be lethal in mice; however, muscle-specific expression of LPL in an LPL -/- background was able to rescue the lethal phenotype [29]. In these crossbred mice, LPL expression is absent in adipose, yet little or no change was noted in adipose tissue mass. It was later shown that normal lipid storage was preserved in these mice by an increase in endogenous fatty acid synthesis [29]. Thus, interpreting the exact role of adipose-derived LPL toward lipid storage in these studies was limited due to compensatory mechanisms that activate endogenous fatty acid synthesis [29]. Because endogenous fatty acid synthesis in adipose is able to counterbalance the loss of adipose-derived LPL activity in vivo, animal studies have not been able to shed light on the quantitative role played by this source of LPL in lipid uptake and storage. In order to directly address this gap in knowledge, we have measured the effect of reducing adipocyte LPL expression on intracellular lipid accumulation using a well-established cultured model of adipocyte differentiation.

## Methods

### Cell culture and adipocyte differentiation

3T3-L1 cells were obtained from American Type Culture Collection (Manassas, VA) and grown in Dulbecco's modified Eagle's medium (DMEM) (Invitrogen, Carlsbad, CA) supplemented with 10% (v/v) fetal calf serum (Irvine Scientific, Santa Ana, CA), 1 mM sodium pyruvate, 0.1 mM non-essential amino acids, 2 mM L-glutamine, 100 μg/ml streptomycin sulfate, and 100 units/ml penicillin. Cells were cultured at 37°C with 10% CO_2 _and passaged twice weekly. To differentiate 3T3-L1 cells into adipocytes, cells were incubated with 250 nM dexamethasone, 450 μM 3-isobutyl-1-methylxanthine, and 167 nM insulin for 2 days, followed by 167 nM insulin for an additional 3 days.

### siRNA Transfection and PCR analysis

Purified Silencer^® ^pre-designed small interfering RNA (siRNA) directed toward mouse lipoprotein lipase (LPL) was synthesized by Ambion Inc. (Austin, TX). The target sequence for the siLPL is on exon 2, with a sense sequence of GCAAAUUUGCCCUAAGGACtt and an antisense sequence of GUCCUUAGGGCAAAUUUGCtt. Cells were transfected with siRNA (100 nM) using DharmaFECT™ 3 transfection reagent according to the manufacturer's specific protocol for 3T3-L1 cells (Dharmacon, Inc., Lafayette, CO). Total RNA was purified from cells using RNeasy (Qiagen, Valencia, CA) and converted to cDNA using TaqMan Reverse Transcriptase (Applied Biosystems, Branchburg, NJ). LPL, leptin, apoCII, apoCIII, and β-actin expression levels were measured by quantitative Real Time PCR analysis (qRT-PCR) of cDNA samples. Primers specific for LPL [GenBank:NM_008509] were designed to amplify 231 base pairs flanking intron 4. Primer sequences for LPL were: upstream, CTGCTGGCGTAGCAGGAAGT; downstream, GCTGGAAAGTGCCTCCATTG. Primers specific for leptin [GenBank:NM_008493] were designed to amplify 213 base pairs flanking intron 2. Primer sequences for leptin were: upstream, TGACACCAAAACCCTCATCA; downstream, TCATTGGCTATCTGCAGCAC. Primers specific for apoCII [GenBank: NM_009695] were designed to amplify 147 base pairs flanking intron 2. Primer sequences for apoCII were: upstream, GCAGGGCTCCCTCTTAAGTT; downstream, AAAATGCCTGCGTAAGTGCT. Primers specific for apoCIII [GenBank: NM_023114] were designed to amplify 99 base pairs flanking intron 3. Primer sequences for apoCIII were: upstream, GGCTGGATGGACAATCACTT; downstream, TGGTTGGTCCTCAGGGTTAG. Primers specific for β-actin [GenBank:NM_007393] were designed to amplify 287 base pairs flanking intron 3. Primer sequences for β-actin were: upstream, CCTGAACCCTAAGGCCAACC; downstream, CAGCTGTGGTGGTGAAGCTG. qRT-PCR was performed using ABsolute QPCR SYBR Green Mix (Fisher Scientific, Atlanta, GA) with the following cycling parameters: 1 cycle, 95°C, 15 min; 40 cycles, 95°C, 15 sec, 60°C, 1 min. β-actin mRNA levels were measured in parallel with identical cycling conditions and used to normalize values obtained for LPL, leptin, apoCII, and apoCIII expression. Relative quantitation was performed using the comparative C_T _method. For this analysis, the amount of target message (LPL, leptin, apoCII, or apoCIII in siRNA-treated adipocytes) was normalized to the internal reference (β-actin) and compared to the calibrator (LPL, leptin, apoCII, or apoCIII in untreated adipocytes).

For straight RT-PCR reactions of leptin, apoCII, apoCIII and β-actin, isolated mRNA was reverse transcribed as described above and cDNA amplification was performed using TaKaRa Ex Taq™ (Fisher Scientific, Atlanta, GA) with the following cycling parameters: 1 cycle, 95°C, 2 min; 40 cycles, 95°C, 30 sec, 60°C, 30 sec, 72 °C 1 min. Amplified products were separated on 3% agarose gels and stained with Gel Star^® ^(Cambrex, Rockland, ME).

### LPL enzymatic activity

Cells, grown in 6-well plates, were rinsed twice with phosphate buffered saline, pH 7 (PBS), and incubated with 200 μg/ml heparin (from porcine intestinal mucosa, 180 USP units/mg, Sigma, St. Louis, MO) prepared in PBS for 15 min at 25°C. Following incubation, the heparin solution was centrifuged (15,000 × g, 5 min, 4°C) to remove cell debris and mixed with an equal volume of 2 mM p-nitrophenyl butyrate (PNPB). Absorbance at 405 nm was recorded following a 10 min incubation using a Genesys UV Spectrophotometer. LPL activity is reported as the amount of p-nitrophenol product formed over the 10 min incubation. The molar extinction coefficient of p-nitrophenol at 405 nm is 11,000 M^-1^cm^-1^.

### Palmitate-albumin complexes

Palmitate (sodium salt), purified bovine LPL, and fatty acid-free albumin were purchased from Sigma. Palmitate-albumin complexes were prepared as follows. Palmitate was dissolved in 3% albumin (wt/v) to a final concentration of 10 mM in 1X Hank's Balanced Salt Solution. The mixture was slowly heated to 50°C and stirred overnight. The mixture was then cooled to 37°C, stirred for an additional 3 hours and sterile filtered through a 0.2 μm syringe filter. Protein was quantified using the BCA™ Protein Assay Kit (Pierce, Rockford, IL).

### Nile Red staining and quantitation

Cells were rinsed twice with PBS, pH 7, and trypsinized with phenol red-free 0.05% Trypsin-EDTA (Invitrogen Corporation, Grand Island, NY) for 5 min at 37°C. Cells were fixed in suspension with 0.5% paraformaldehyde (v/v) in PBS for 10 min at 25°C. A stock solution of 1 mg/ml Nile Red (Sigma, St. Louis, MO) was prepared in acetone and added to the cell preparation to a final concentration of 5 μg/ml. Cells were incubated at 25°C for 30 min in the dark. Flow cytometry was performed using a FACScan flow cytometer (λ_excitation _488 nm; λ_emission _585 nm). Nile Red fluorescence was quantitated using a Shimadzu RF-1501 spectrofluorophotometer (λ_excitation _488 nm; λ_emission _560 nm).

### Statistical analysis

All experimental protocols were done in triplicate. All error bars represent standard deviation of triplicate points.

## Results

### LPL expression and cell-surface enzymatic activity of 3T3-L1 preadipocytes and differentiated adipocytes

**Figure 1:**
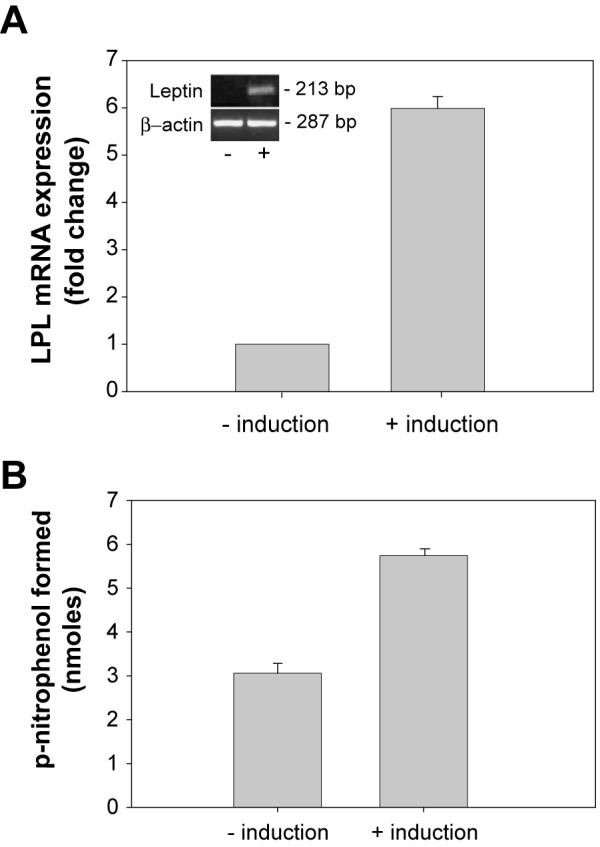
**LPL gene expression and enzymatic levels increase following differentiation of pre-adipocytes**. 3T3-L1 cells were treated with or without adipocyte induction reagents as described in Methods. (A) Total RNA was extracted from cells and LPL mRNA levels were measured by qRT-PCR using the Comparative CT method. Inset, specific amplification of leptin sequence (213 bp) was done by straight reverse transcriptase-PCR to confirm differentiation of pre-adipocytes. Amplification of β-actin sequence (287 bp) was performed as a loading control. (B) Cells were incubated with heparin (200 μg/ml) to release surface associated LPL. The heparin releasable fraction was then incubated with p-nitrophenyl butyrate (1 mM final concentration) for 10 min and absorbance was measured at 405 nm.

**Figure 2:**
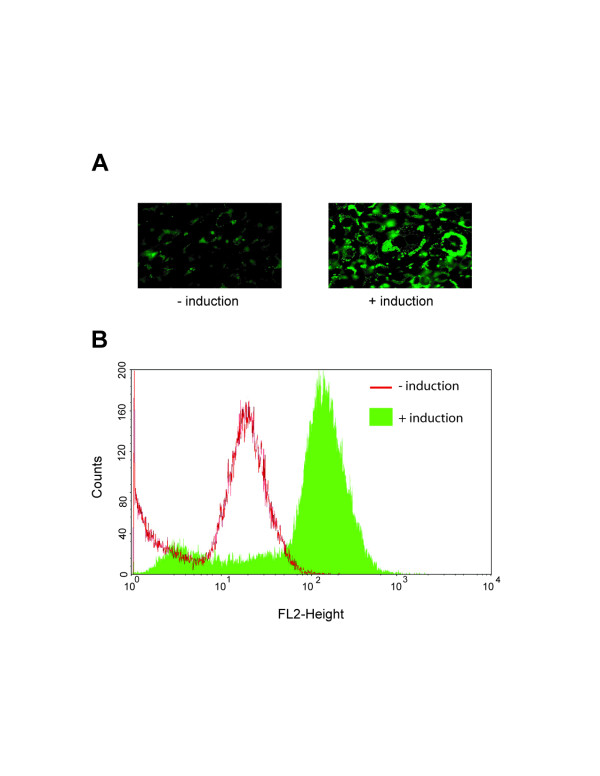
**Intracellular lipid accumulation during differentiation of pre-adipocytes correlates with increased LPL expression**. 3T3-L1 cells, treated with or without adipocyte induction reagents, were incubated with Nile Red (5 μg/ml). Lipid associated Nile Red was then viewed by fluorescence microscopy (630X) and quantified by flow cytometry.

Although it is known that adipocytes synthesize LPL in vivo, identifying the quantitative changes in LPL transcription during adipocyte differentiation have only been partially examined [30] and no information is available as to the relationship between changes in LPL mRNA levels and LPL enzymatic activities. To examine this relationship, we induced differentiation in 3T3-L1 mouse pre-adipocytes with a standard cocktail of dexamethasone, 3-isobutyl-1-methylxanthine, and insulin, and measured LPL transcriptional changes by quantitative qRT-PCR and LPL enzymatic activity by a colorimetric assay. Upon differentiation, LPL mRNA levels increased approximately 6-fold, which is in close agreement with prior measurements [26] (Fig. 1A). To measure cell surface-associated LPL enzymatic levels, cells were incubated with heparin to release HSPG-bound LPL and this material was incubated with lipase-specific substrate p-nitrophenyl butyrate. We found that LPL enzymatic levels increased 2-fold following adipocyte differentiation (Fig. 1B). Full differentiation of these cells was confirmed by activation of leptin expression (Fig. 1A, inset). We also stained differentiated adipocytes in parallel with the fluorescent lipophilic dye, Nile Red, and quantitated increases in intracellular lipid storage by flow cytometry (Fig. 2). Intracellular lipids increased by ~10-fold demonstrating a direct correlation between LPL expression and lipid storage.

### LPL expression by adipocytes is necessary for intracellular lipid accumulation

**Figure 3:**
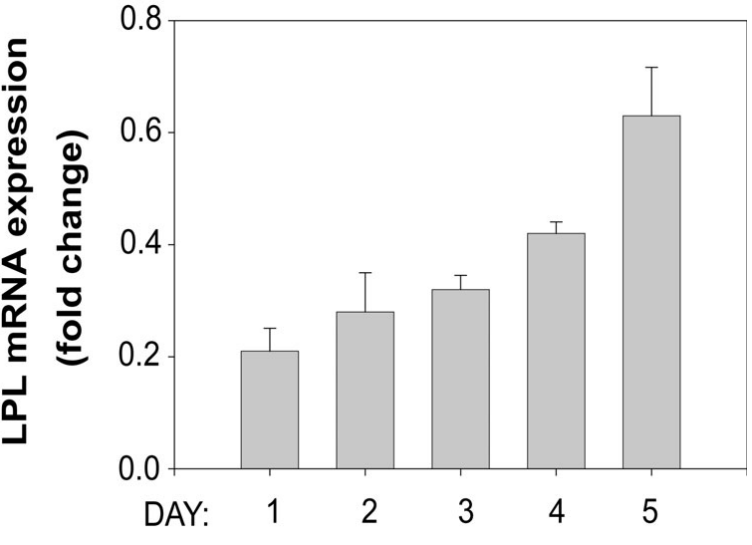
**siRNA-mediated reduction in LPL expression over a 5 day adipocyte differentiation period**. 3T3-L1 cells were transfected with siRNA specific for LPL, followed by incubation with adipocyte induction reagents. After each 24 h period up to day 5 post-induction, total RNA was isolated from adipocytes and LPL expression was assessed by qRT-PCR. Results were analyzed by the Comparative C_T _method with fully differentiated, non-treated adipocytes representing 100% LPL expression. β-actin expression was amplified in parallel and used to normalize replicate points.

As described above, attempts to measure the contributions of LPL to lipid storage in adipocytes in vivo have been met with difficulty due to activation of endogenous fatty acid synthesis. This activation is largely due to hormonal regulation of adipocyte physiology [3] and also to what has been attributed to an organismic survival function [29,31]. Therefore, the most straightforward approach to determine if adipocyte-derived LPL directly contributes to lipid uptake and storage is to examine the affects of inhibiting LPL expression on lipid accumulation using cultured adipocytes. Quantifying intracellular lipid accumulation is easily accomplished by measuring incorporation of Nile Red by fluorometry as shown in Fig. 2. In order to obtain measurable Nile Red fluorescence, pre-adipocytes must be allowed to take up and store lipids for the full 5 days of differentiation. We chose to use siRNA methodologies to inhibit LPL expression; however, because the transfection process might adversely affect critical events during differentiation, we chose to perform our siRNA transfections prior to initiating adipocyte induction. For this approach to work, our siRNA treatment would have to inhibit LPL expression for a minimum of 5 days rather than the typical 24–48 hour period in order to accommodate the minimum time necessary for full differentiation. To determine if siRNA treatment will be able to sufficiently inhibit LPL expression for 5 days following transfections, we performed a time course study measuring LPL expression by qRT-PCR during the entire course of differentiation. 3T3-L1 pre-adipocytes were transfected with LPL specific siRNA, allowed to recover for the prescribed 3 h, and incubated with adipocyte induction reagents. At 24 h intervals up to 5 days, total RNA was extracted from cells and subjected to qRT-PCR analysis to measure siRNA-mediated inhibition of LPL expression. Twenty four hours post-transfection, we found LPL mRNA expression inhibited by 80% (Fig. 3). LPL expression showed a steady recovery after each subsequent 24 hour period; yet by day 5, our endpoint for quantitating lipid accumulation, LPL expression was still inhibited by ~40%.

**Figure 4:**
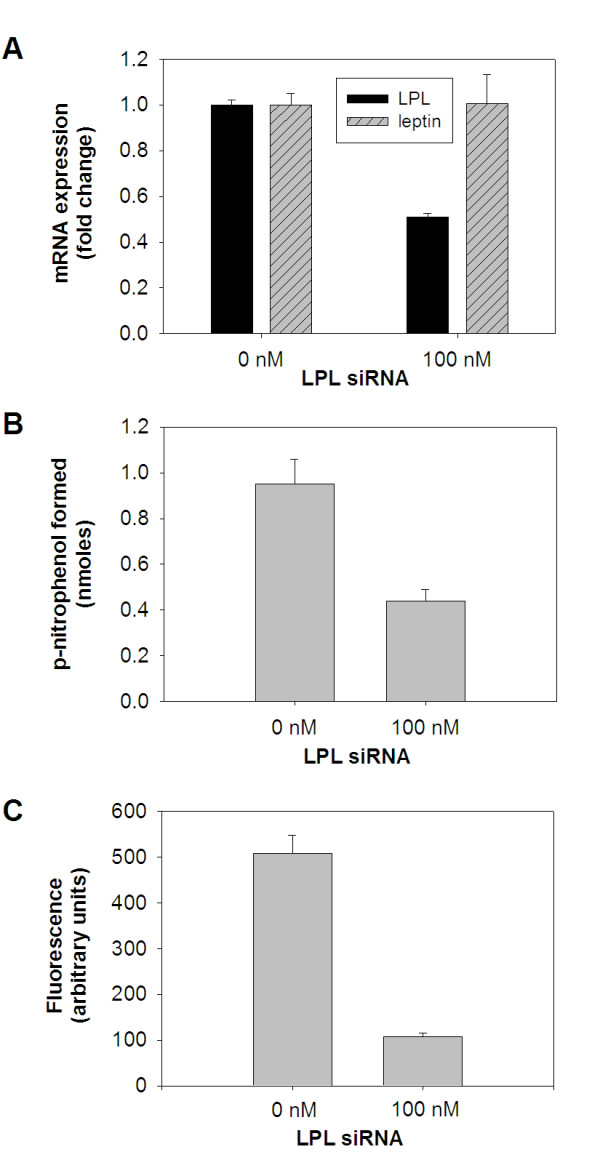
**Inhibition of LPL gene expression in adipocytes causes a corresponding reduction in cell surface, HSPG-associated LPL activity and intracellular lipid accumulation**. 3T3-L1 cells were transfected with LPL-specific siRNA, followed by incubation with adipocyte induction reagents for 5 days. (A) Total RNA was isolated from cells and subjected to qRT-PCR analysis to determine relative LPL and leptin mRNA expression levels in siRNA treated and non-treated control cells. (B) Heparin-releasable LPL enzymatic activity was measured in siRNA treated and non-treated control cells as described in Fig. 1. (C) Intracellular lipid droplet formation was quantified by measuring adipocyte-associated Nile Red using fluorometry.

Based on these findings we predicted that the level of inhibition measured after 5 days would be sufficient to determine if endogenously synthesized LPL contributed to lipid accumulation in our cultured adipocyte model. We transfected 3T3-L1 pre-adipocytes with LPL-specific siRNA, induced adipocyte differentiation for 5 days, and then examined 1) LPL mRNA expression changes due to siRNA treatment, 2) the resulting affect on cell surface LPL activity levels, and 3) performed a quantitative evaluation of intracellular lipid accumulation (Fig. 4). Similar to the results we obtained for Fig. 3, siRNA treatment reduced LPL mRNA expression to ~50% of non-treated controls after the 5 day adipocyte induction period (Fig. 4A). Importantly, siRNA treatment showed no effect on adipocyte differentiation as no measurable change was found for leptin expression when comparing siRNA treated versus non-treated cells (Fig. 4A). Cell surface associated, heparin-releasable LPL enzymatic activity was also reduced by ~50% in siRNA treated cells (Fig. 4B). Most importantly, Nile Red staining of siRNA treated adipocytes was reduced by 80% compared with non-treated cells (Fig. 4C).

### Exogenous addition of LPL or palmitate restores intracellular fat storage in siRNA treated adipocytes

**Figure 5:**
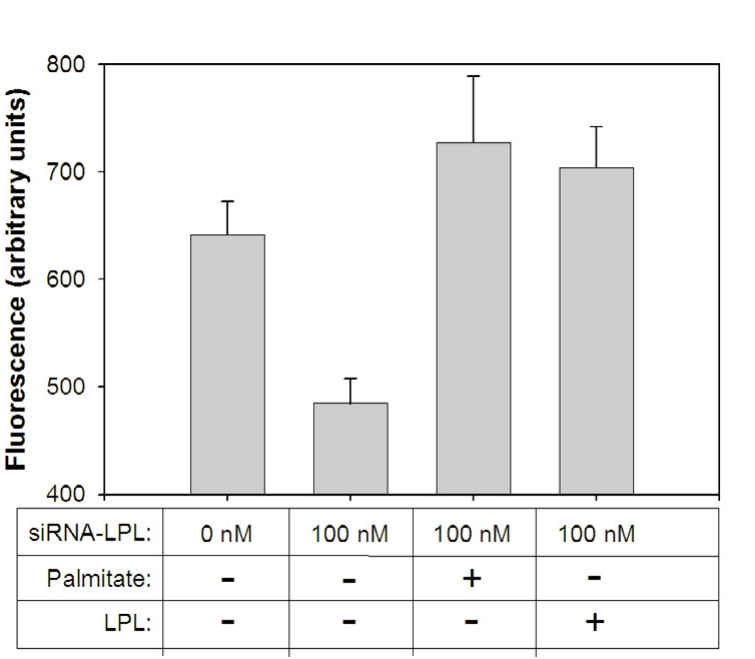
**Exogenous addition of LPL or palmitate reverses the effect of LPL-siRNA treatment on lipid accumulation by adipocytes**. 3T3-L1 cells were transfected with or without LPL-specific siRNA (100 nM), followed by incubation with adipocyte induction reagents. In parallel, some cells were also incubated with palmitate-albumin (2 mg/ml protein) beginning with the second addition of insulin during the differentiation protocol. Alternatively, LPL (6.67 Units/ml) was added with the second addition of insulin and each successive day until measuring intracellular lipid levels. After a total of five days, intracellular lipid accumulation was quantified by measuring cell-associated Nile Red using fluorometry.

Since siRNA-mediated inhibition of LPL in adipocytes significantly reduced intracellular lipid storage, we reasoned that the exogenous addition of purified LPL (to restore extracellular lipase activity lost due to siRNA treatment) or palmitate (to supply cells with a source of available free fatty acid thus abrogating the need for LPL hydrolysis activity) would be expected to reverse the phenotype seen in siRNA treated cells. To establish control values, adipocytes were treated with or without siRNA for LPL and intracellular lipid accumulation was quantified by Nile Red staining as was done in Figure 4C. In parallel, siRNA-treated adipocytes were also incubated with either purified LPL or palmitate which was pre-bound to albumin to serve as a soluble fatty acid carrier. As shown in Figure 5, both of these additions were independently able to reverse the effects of reducing LPL expression by restoring intracellular lipid to levels obtained in non-siRNA-treated cells. These results complement the data acquired from simple siRNA treatment and provide additional evidence that adipocyte-derived LPL activity is essential to generate available fatty acids for uptake and storage.

### ApoCII and apoCIII expression in adipocytes

**Figure 6:**
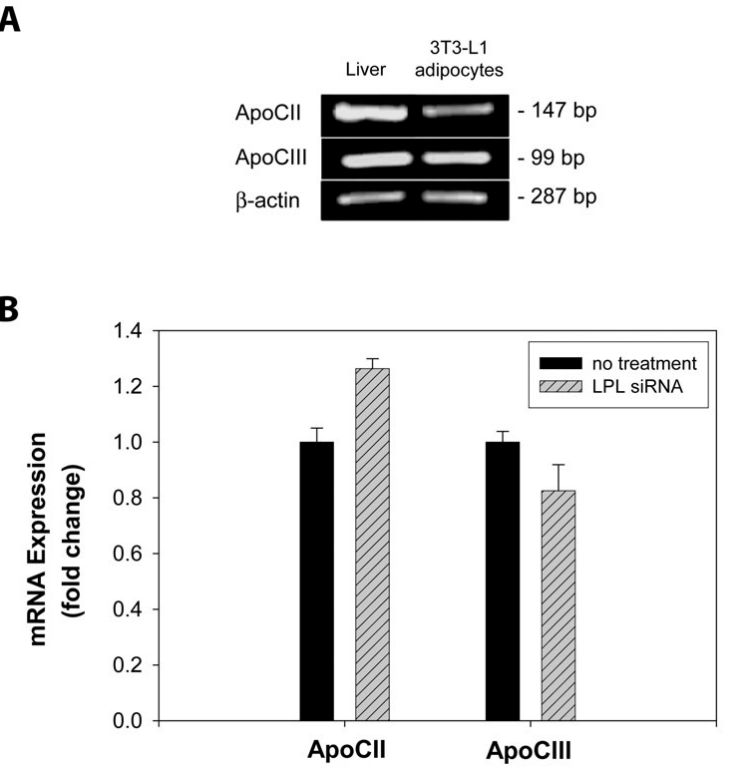
**Expression of apoCII and apoCIII in control versus LPL-siRNA-treated adipocytes**. (A) 3T3-L1 pre-adipocytes were incubated with induction reagents for 5 days as in Fig. 1. Total RNA was isolated from 3T3-L1 adipocytes and mouse liver. mRNA was converted to cDNA with reverse transcriptase and the sequences for apoCII (147bp) and apoCIII (99 bp) were amplified by PCR. Amplification of β-actin sequence (287 bp) was performed as a loading control. (B) 3T3-L1 cells were transfected with LPL-siRNA, followed by incubation with adipocyte induction reagents. After five days post-induction, total RNA was isolated from adipocytes and apoCII and apoCIII expression were assessed by qRT-PCR. Results were analyzed by the Comparative CT method, with fully differentiated, non-treated adipocytes representing 100% expression. Amplification of β-actin mRNA was performed in parallel and used to normalize replicate points.

ApoCII and apoCIII are known to be, respectively, a strong activator and strong inhibitor of LPL [32,33]. Within the vasculature, apoCII and apoCIII are readily available to regulate LPL activity since they are found associated with circulating lipoprotein particles [34,35]. Although the primary in vivo site for synthesis of apoCII and apoCIII is the liver [36], we questioned if adipocytes are also able to synthesize their own source of apoCII and apoCIII which would provide these extravascular cells the means to regulate LPL activity independent of circulating apoCII and apoCIII. To date, expression of these LPL regulators by adipocytes has not been examined. To address this question, we first performed RT-PCR analysis on cDNA samples generated from differentiated 3T3-L1 adipocyte RNA. As shown in Figure 6A, cultured adipocytes do in fact express both apoCII and apoCIII.

Since apoCII and apoCIII have opposite effects on LPL activity, it is generally accepted that the state of LPL activity depends on the ratio of apoCII/apoCIII expression. A high ratio of apoCII/apoCIII promotes LPL activity, whereas a low apoCII/apoCIII ratio inhibits LPL function. Since this ratio can significantly affect extracellular LPL activity and in turn fatty acid uptake and storage by adipocytes, we determined apoCII and apoCIII expression levels in adipocytes treated with or without siRNA for LPL. Although expression of both regulators was largely unaffected by LPL-siRNA treatment, we did find a small but reproducible effect on their expression as a result of diminished LPL expression (Fig. 6B). ApoCII expression increased by ~20%, while apoCIII expression decreased by ~20%.

## Discussion

The generation of fatty acids from dietary sources and their uptake by cells are essential processes for efficient energy metabolism and storage. A general role for LPL in these processes has been confirmed from LPL gene ablation studies in mice which results in a lethal phenotype [28]. Naturally occurring LPL gene mutations have also been reported in the human population that lead to severe hypertriglyceridemia [37]. However, in light of these studies which have established a critical systemic role for LPL in overall fatty acid handling, investigations carried out to evaluate the direct contributions of adipocyte-specific LPL toward lipid storage have not yielded conclusive evidence because of compensatory increases in endogenous fatty acid synthesis [29]. The resulting limitations of published in vivo studies are likely due to hormonal regulation responsible for adipose mass preservation [29]. To circumvent this problem and obtain direct quantitative evidence of the role of adipocyte-derived LPL in lipid storage, we used a well established in vitro culture model of adipocyte differentiation [38]. Differentiation of this culture model to full adipocytes resulted in a 6-fold increase in LPL mRNA expression. This increase in LPL mRNA translated into a 2-fold increase in cell surface-associated LPL enzymatic activity and, importantly, ~10-fold increase in intracellular lipid storage. Using siRNA specific for LPL, we were able to reduce LPL expression by ≥ 50% during the course of adipocyte differentiation. This reduction in adipocyte LPL expression resulted in a parallel decrease in lipid storage of ~80%, which was completely reversed by the exogenous addition of either purified LPL or palmitate. These results clearly establish that adipocyte-derived LPL plays a quantitatively important role in intracellular lipid accumulation.

It is now well established that LPL requires apoCII as a co-factor for efficient triacylglycerol hydrolysis [39,40]. This fact is highlighted by the observation that mutations in the apoCII gene lead to abnormally high levels of circulating triacylglycerols [41]. Following its synthesis and secretion by hepatocytes, apoCII associates with circulating lipoproteins and stimulates LPL-mediated hydrolysis once in contact with LPL that is associated with the vascular wall. ApoCII is a multidomain protein with binding sites for both LPL and lipid components of circulating lipoprotein particles [42,43]. The dual binding domains of apoCII are thought to anchor LPL to the particle surface while permitting substrate access to its active site [42]. In contrast, apoCIII is a potent inhibitor of LPL activity [44,45]. Because of this, the expression levels, or ratio, of apoCII to apoCIII can be a major influence on extracellular LPL activity [46]. ApoCII and apoCIII are known to carry out their functions in the circulation largely while bound to lipoproteins. In the extravascular environment, we questioned if other non-hepatic sources, such as adipocytes, contribute to expression of apoCII and apoCIII and in doing so provide their own source of LPL regulators to manage extravascular lipid hydrolysis. This is indeed the case for differentiated adipocytes as RT-PCR analysis revealed expression of both apoCII and apoCIII. We also examined the apoCII/apoCIII expression ratio by qRT-PCR analysis and found potential regulation of this ratio in adipocytes that appears to be influenced by LPL expression levels; decreased LPL expression following siRNA treatment resulted in a small but reproducible increase in apoCII and decrease in apoCIII expression. This change in the apoCII/apoCIII ratio may reflect a compensatory mechanism whereby reduced levels of LPL are counterbalanced by an increase in the apoCII/apoCIII ratio. Increasing the apoCII/apoCIII ratio may serve to ensure more effective activation of the remaining pool of extracellular LPL.

## Competing interests

The author(s) declare that they have no competing interests.

## Authors' contributions

AMG carried out the experiments in this study and participated in the experimental design. RAO provided the original conceptual framework for the study, assisted with the experimental design and finalized the manuscript for submission. All authors read and approved the final version.
